# Fabrication and Characterization of Electrospun Folic Acid/Hybrid Fibers: In Vitro Controlled Release Study and Cytocompatibility Assays

**DOI:** 10.3390/polym13203594

**Published:** 2021-10-19

**Authors:** Fatma Nur Parın, Sana Ullah, Kenan Yıldırım, Motahira Hashmi, Ick-Soo Kim

**Affiliations:** 1 Faculty of Engineering and Nature Science, Department of Polymer Materials Engineering, Mimar Sinan Campus, Bursa Technical University, Bursa 16310, Turkey; kenan.yildirim@btu.edu.tr; 2 Nano Fusion Technology Research Group, Division of Frontier Fibers, Institute for Fiber Engineering (IFES), Interdisciplinary Cluster for Cutting Edge Research (ICCER), Shinshu University, Ueda 386-8567, Japan; sanamalik269@gmail.com (S.U.); motahirashah31@gmail.com (M.H.)

**Keywords:** hybrid nanofiber, cytotoxicity, folic acid, in vitro study, drug release

## Abstract

The fabrication of skin-care products with therapeutic properties has been significant for human health trends. In this study, we developed efficient hydrophilic composite nanofibers (NFs) loaded with the folic acid (FA) by electrospinning and electrospraying processes for tissue engineering or wound healing cosmetic applications. The morphological, chemical and thermal characteristics, in vitro release properties, and cytocompatibility of the resulting composite fibers with the same amount of folic acid were analyzed. The SEM micrographs indicate that the obtained nanofibers were in the nanometer range, with an average fiber diameter of 75–270 nm and a good porosity ratio (34–55%). The TGA curves show that FA inhibits the degradation of the polymer and acts as an antioxidant at high temperatures. More physical interaction between FA and matrices has been shown to occur in the electrospray process than in the electrospinning process. A UV-Vis in vitro study of FA-loaded electrospun fibers for 8 h in artificial acidic (pH 5.44) and alkaline (pH 8.04) sweat solutions exhibited a rapid release of FA-loaded electrospun fibers, showing the effect of polymer matrix–FA interactions and fabrication processes on their release from the nanofibers. PVA-CHi/FA webs have the highest release value, with 95.2% in alkaline media. In acidic media, the highest release (92%) occurred on the PVA-Gel–CHi/sFA sample, and this followed first-order and Korsmeyer–Peppas kinetic models. Further, the L929 cytocompatibility assay results pointed out that all NFs (with/without FA) generated had no cell toxicity; on the contrary, the FA in the fibers facilitates cell growth. Therefore, the nanofibers are a potential candidate material in skin-care and tissue engineering applications.

## 1. Introduction

Nanofibers have played a key role in different areas of biomedical research, ranging from drug release to wound healing, and cell regeneration owing to their high specific surface area and capacity to adjust their properties by altering composition and production conditions [1,2,3,4]. The use of hydrophilic polymers in the fabrication of electrospun nanofibers has been proven to be beneficial in the development of fast-dissolving delivery systems with decreased drug–drug interactions [5,6,7]. Generally, a blended combination of natural and synthetic polymers is preferred. Synthetic polymers can offer proper mechanical strength while also enhancing electrospinnability. Natural polymers can promote cellular adhesion and thus better imitate the ECM environment [8]. Furthermore, the increased hydrophilicity supplied by natural polymers is favorable when carrying hydrophilic drugs. Nanofibrous scaffolds made of natural and synthetic polymers were shown to be suitable for designing drug release systems via the electrospinning of blended polymers or combination with bioactive agents [9,10,11,12,13,14]. Previously, electrospun nanofibers have been produced from single polymer sources, and more recently, polymer combinations have been created to develop so-called polyblend nanofibers. Polyblend nanofibers are a new type of biomaterial that can function as mimics for native tissue while presenting topographical and biochemical stimuli that support healing [15,16,17].

Scaffolds are made from a variety of polymers, including poly(vinyl alcohol), poly(ethyl oxide), poly(lactic acid), poly(glycolic acid), poly(lactic-co-glycolic acid), poly(caprolactone), and natural extracellular matrix (ECM) analogs, such as collagen, gelatin, silk, chitin, chitosan, alginate, and hyaluronic acid, because of their lack of toxicity, superior biocompatibility, cell adhesion, and proliferation capability [18]. In this context, hydrophilic PVA-based materials can be blended with natural polymers and utilized in drug delivery systems [18,19,20,21,22,23,24].

Bioactive compounds can partially lose their therapeutic efficiency during the blend–electrospinning process. It is critical to have protective agents. To ensure that drugs stay bioactive, certain procedures must be performed [8]. In this context, electrospraying can be utilized for drug release applications, especially the encapsulation of bioactive compounds [25]. Electrospraying, also referred to as electro hydrodynamic atomization (EHDA), is usually presented as a unique and simple technology capable of controlling droplet formation electrostatically [26]. Folic acid (FA) is defined as a multifunctional, active substance that is safe to use and even combine with other chemicals [27]. Moreover, folic acid has received much attention from researchers in the fields of biomedical, bioengineering, and regenerative therapies, because of its non-immunogenicity, high stability, and ability to support tissue regeneration/repair [26,27]. In this regard, an increasing number of studies on the use of folic acid in micro/nanocapsule, liposome, hydrogel and/or nanofiber forms are available in a wide range of applications [28,29,30]. However, there are no studies in the literature on the utilization of nanofibers containing FA in tissue engineering applications, formed by both blending electrospinning and simultaneous (electrospinning and electrospraying) processes.

The main objective of this study is to fabricate various PVA-based nanofibers, including FA, via two distinct processes, electrospinning and simultaneous spinning (electrospinning and e-spraying), and then to evaluate their controlled release and cell growth characteristics. The properties of PVA-based NFs in terms of morphology, thermal resistance and chemical structure were analyzed by changing the type of polymer matrix. In addition, we assessed which had better cell growth and release values.

## 2. Materials and Methods

### 2.1. Materials

The Poly(vinyl alcohol) (PVA) (density 0.4–0.6 g/cm^3^, purity 87.8% and M_w_~30.000 g/mol) was purchased from ZAG Industrial Chemicals (Istanbul, Turkey). Gelatin from bovine skin (225 Bloom, type B, Bio Reagent) was supplied from Sigma Aldrich Chemical Company (St. Louis, MO, USA). Sodium alginate (medium molecular weight) and sodium bicarbonate (NaHCO_3_) (99.7% purity) were purchased from Sigma Aldrich Chemical Company (St. Louis, MO, USA). Acetic acid (CH_3_COOH) with 80% purity was also obtained from Sigma Aldrich Chemical Company (St. Louis, MO, USA), and ethanol (99.9% purity) was purchased from Tekkim Chemical Company (Bursa, Turkey). Chitosan (M_n_ 100,000–300,000) was supplied by Alfa Aesar Fine Chemicals and Metals (USA). Folic acid (C_19_H_19_N_7_O_6_) for biochemistry (98–102% purity) was provided by ChemSolute Company (Germany). The ethanol (99% purity) was purchased from Sigma Aldrich Chemical Company (St. Louis, MO, USA). l-Histidine mono-hydrochloride monohydrate (C_6_H_9_O_2_N_3_.HCI.H_2_O) (>99% purity), sodium chloride (>99.5% purity), and sodium dihydrogen phosphate dihydrate (NaH_2_PO_4_.2H_2_O) were supplied by Sigma Aldrich Chemical Company (St. Louis, MO, USA) to produce the artificial sweat solutions.

### 2.2. Methods

#### 2.2.1. Fabrication of Neat Nanofibers and FA-Loaded Hybrid Nanofiber via Blending Electrospinning

Polymer solutions were prepared as in the previous study [10]. PVA granules were dissolved in distilled water at 90 °C until a 10% (*w*/*w*) homogenous PVA solution was produced. By stirring for 2 h at room temperature, gelatin was dissolved in distilled water and acetic acid binary-solvent systems (7:3 *w*/*w*) solution to get a 20% (*w*/*w*) gelatin solution. Alginate powders were dissolved in water to make a 2% (*w*/*w*) alginate solution. Chitosan powders were dissolved in acetic acid and distilled water binary-solvent systems (9:1 *w*/*w*) solution to obtain 7% chitosan solution. The process parameters and features of the composite polymer solutions are shown in Table 1 below. The average temperature was 25 °C, the % humidity was lower than 60, and the speed of the rotating collector was 150 rpm. In all FA-loaded polymer solutions, 22 mg of FA in 1 M 1 mL of NaHCO_3_ was homogeneously added to the blend solutions.

**Figure 1:**
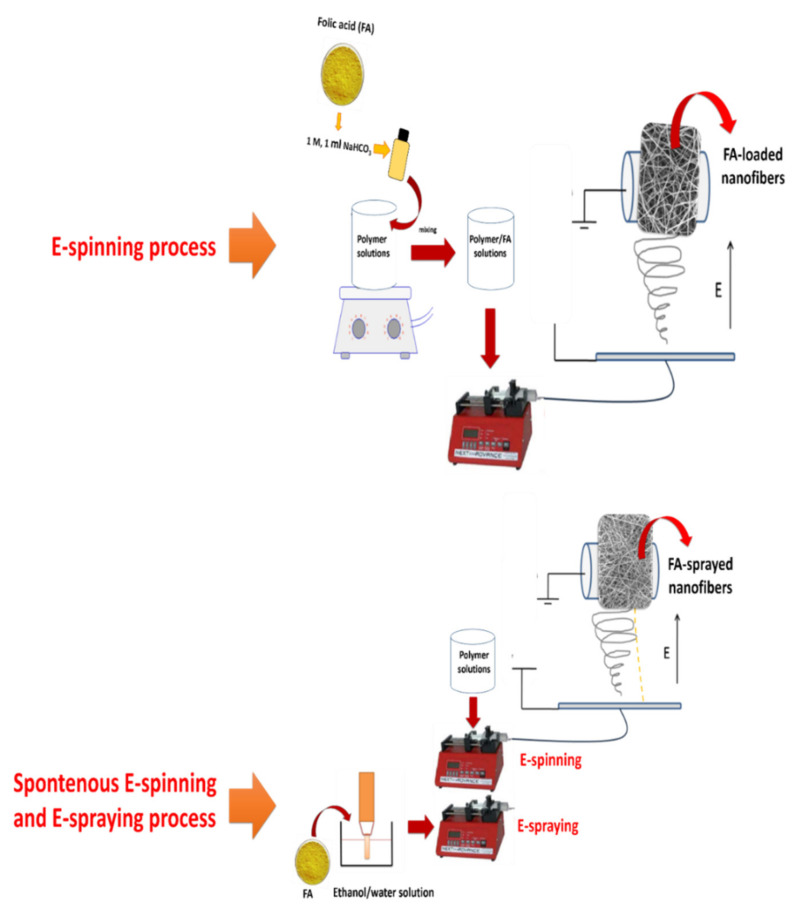
Schematic illustration of the processes.

The electrospinning of PVA-based composite nanofibers and FA-loaded PVA-based composite nanofibers was carried out by an electrospinning process (INOVENSO Nanospinner24 Device, Turkey). Neat polymer solutions without FA were prepared as a control. In the electrospinning process, the polymer solutions were transferred into plastic syringes (10 mL) and these syringes were attached to stainless nozzles with 0.5 mm inner diameters (Figure 1). Polymer solutions were dispensed into the rotary collector. Finally, neat and FA-loaded nanofibers were deposited in the collector due to the high electrical voltage.

#### 2.2.2. Fabrication of FA-Spraying on Hybrid Nanofibers via Simultaneous Process

The simultaneous process (electrospinning + e-spraying process) has been detailed in a previous study [10]. Two syringe pumps for polymer solution and FA dispersion were utilized in the experiments. The environmental factors and the speed of the collector were the same as in blending electrospinning process. Schematic illustrations of the two distinct processes are shown in Figure 1. In the simultaneous process, all polymer solutions were prepared. Afterwards, the sonicated FA–ethanol solution and the polymer solutions were transferred into two distinct plastic syringes. These solutions were fed into the system by two distinct syringe pumps as in the electrospinning process.

The samples are encoded as polymer blend/FA and polymer blend/sFA for the electrospinning process and simultaneous process (electrospinning + e-spraying process), respectively.

#### 2.2.3. Microstructural Analysis of the Hybrid Nanofibers

A Carl Zeiss/Gemini 300 Scanning Electron Microscope (SEM) was used to examine the morphology of all nanofibers (ZEISS Ltd., Jena, Germany). Before analysis, all samples were coated with gold for 20 min. The diameters of 60 individual fibers were randomly selected for each sample and measured using ImageJ (version 1.520 software). 

#### 2.2.4. Physical Analysis of Nanofibers

The ATR-FTIR spectra of nanofibers were acquired using a ThermoNicolet iS50 FT-IR (USA) spectrometer and an ATR (Attenuated Total Reflectance) adaptor (Smart Orbit Diamond, MI, USA). Spectra in the range of 500–4000 cm^−1^ with automatic signal gain were collected (16 scans) at a 4 cm^−1^ resolution

#### 2.2.5. Thermal Analysis of Nanofibers

The thermal analysis was performed using a TA/SDT650 TGA (USA). TGA analysis was carried out in a nitrogen (N_2(g)_) atmosphere with a 20 °C min^−1^ heating rate and a temperature range of 30–600 °C, and in an oxygen (O_2(g)_) atmosphere with a 20 °C min^−1^ heating rate and a temperature range of 600–900 °C.

#### 2.2.6. Drug Release of Nanofibers and Kinetics

The ISO 105-E04:2013 procedure [31] was used to make the artificial sweat solutions. The total immersion procedure was performed to evaluate the vitamin-release behavior of FA-sprayed final fibers in acidic and alkaline sweat solutions at pH 5.44 and pH 8.04 [31]. Nanofibers of 30 cm^2^ were placed in sealed glass tubes with 100 mL of acidic and alkaline sweat solution, respectively. After this, they were placed in a shaking incubator at 36 °C with 200 rpm shaking, and we applied the folic acid release profile. Samples of 3.5 mL were taken of the sweat solutions at the specified time intervals, and the associated absorbance value was determined in a UV-Vis spectrophotometer (Scinco/NEOYSY 2000, Seoul, South Korea) at max = 282 nm, which is folic acid’s typical peak. The drug concentrations were determined using the calibration curve of a model vitamin prepared with a known concentration of folic acid in acidic and alkaline solutions. For the acidic solution, the calibration curve was calculated to be Y = 0.0486X + (−0.0402) (R^2^ = 0.99992), and for alkaline solution, Y = 0.0551X + (−0.0422) (R^2^ = 0.99959), where X is the FA concentration (mg/L) and Y is the solution absorbance at 282 nm (linear range of 0.5–25 mg/L). UV-Vis spectroscopy was used to determine the amount of drug released. For each experiment, three replicates were obtained.

The kinetics of drug release were substantially controlled by polymer blends. The main mechanisms for drug release from a polymer matrix include drug diffusion and polymer relaxation/dissolution [32]. The cumulative release of FA (%) from all nanofibers was plotted with time. In this study, the kinetic mechanisms of all obtained nanofibers were calculated with zero-order, first-order, Hixson Crowell, Higuchi, and Korsmeyer–Peppas mathematical models. The different kinetic models are shown in Table 2.

#### 2.2.7. Entrapment Efficiency (EE) and FA Loading Capacity (LC) in Nanofibers

The FA entrapment efficiency (EE, %) was calculated as follows.

A previously known area of the fibrous materials (3 × 3 cm^2^) was completely dissolved separately in acidic and alkaline sweat solutions. Afterward, about 3 mL of these solutions was evaluated at the FA characteristic wavelength using UV-Vis spectroscopy. The FA loading capacity of the nanofibers (LC, %) was measured to specify how much FA per polymer was entrapped in them. These equations are shown as Equations (1) and (2).

(1) $$\left(100-EE\right)\%=\frac{Amount\;of\;total\;FA\;release-Unentrapped\;FA\;amount}{\mathrm{Amount}\;\mathrm{of}\;\mathrm{total}\;\mathrm{FA}\;}\times 100$$

(2) $$\mathrm{LC}=\frac{\mathrm{Amount}\;\mathrm{of}\;\mathrm{total}\;\mathrm{FA}\;\mathrm{release}}{\mathrm{Initial}\;\mathrm{amount}\;\mathrm{of}\;\mathrm{FA}\;\mathrm{containing}\;\mathrm{nanofiber}}\times 100$$

#### 2.2.8. Cytotoxicity of Nanofibers

##### Preparation of Cell Culture

In the cell culture laboratory of the Biotechnology Department of the Bursa Technical University, all experimental aspects of the cell culture and cytotoxicity assessments were carried out in accordance with acceptable cell culture practices (ISO Standard) [32]. The L929 cell lines (mouse fibroblasts), as a reference cell line of the UNI EN ISO 10993-5 rule: 2009, were used to test medical products and materials for cytotoxicity [33]. L929 cells were seeded in T75 bottles with the addition of 10% FBS and 1% penicillin streptomycin to RPMI 1640. The cells were cultured in a humidified cell culture incubator at 37 °C in 5% CO_2_ and observed daily with an inverted microscope and phase contrast (Olympus CKX41). When 80% confluence was detected, subculturing was carried out. In 96 tissue culture wells, a total of 5 cells/well were placed following disaggregation with trypsin/EDTA and the resuspension of cells in the media.

##### Preparation of Nanofiber Extract Solutions and MTT Assays

The ISO 10993-12: 2009 standard [34] was utilized in this study for the sample preparation of both the reference and the new nanofiber material. Both sides of standard-sized samples (3 cm^2^ /mL) were sterilized in a laminar flow cabinet for 1 h under a UV light. In order to obtain extract solutions, 30 cm^2^ (6 cm × 5 cm) sterile samples were placed in a 10 mL culture medium (RPMI 1640 with 1% Penicillin–Streptomycin, 10% serum) and cultured at 37 °C for 1 h. The samples were removed at the end of the extraction period, and the extract solutions were held at 37 °C during the cytotoxicity testing. MTT analysis was performed to determine the cytotoxicity of nanofiber extract solutions in the L929 cell line. In this regard, cells were exposed to the nanofiber extract solutions after 24 h of incubation of the cells seeded in 96-well tissue culture plates. Then, the media were aspirated to add MTT (5 mg/mL of stock of PBS) and the cells were incubated with MTT dye for a further 4 h (10 mL/well in 100 mL of cell suspension). Finally, the dye was carefully removed and 100 mL DMSO was added to each well. The absorption of the solution in each well was evaluated within a microplate reader at 570 nm. To assess cytotoxicity, the effects of 100% concentrations of original extract solutions from all reference and novel nanofiber samples on treated cells were compared to a negative control group that did not receive any chemicals. The average absorbance values and standard deviation values of living cells were calculated by averaging all the data obtained. Furthermore, cell viability in the control group was assumed to be 100%, and live cell percentages were assessed for all sample groups in comparison to the control.

### 2.3. Statistical Analysis

Statistical analysis of % cell viability values obtained after the cytocompability experiments were performed using IBM SPSS Statistics version 22 software (IBM software, Armonk, NY, USA). The results were statistically analyzed by Student–Newman–Keuls (SNK) test to determine the importance of the difference between different groups. The significance value level was identified as *p* < 0.05.

## 3. Results and Discussion

### 3.1. Morphology of the Hybrid Nanofibers

**Figure 2:**
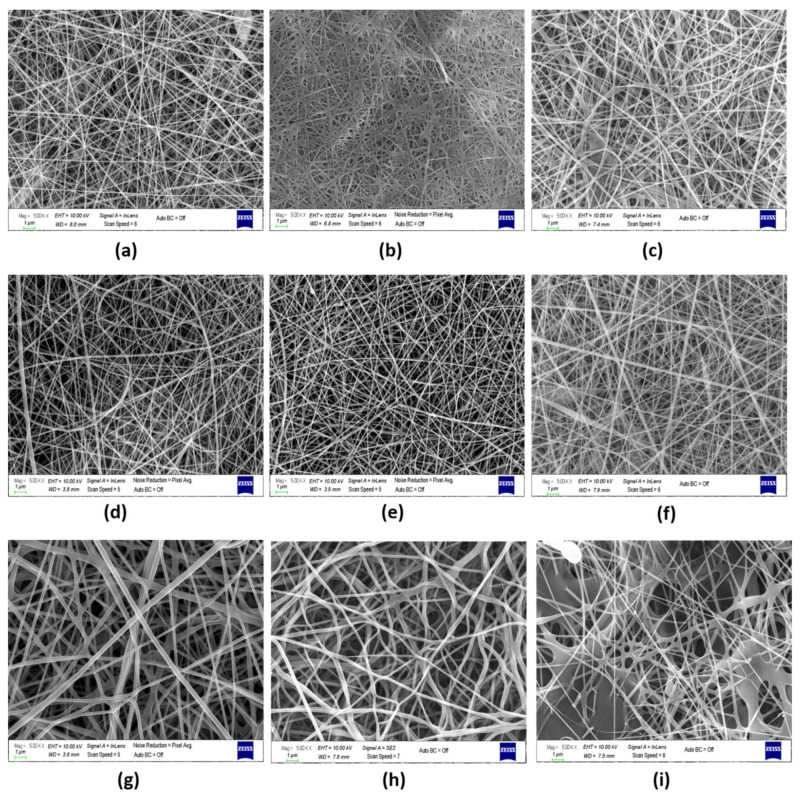
(**a**) SEM image of PVA-Gel, (**b**) PVA-Gel/FA, (**c**) PVA-Gel/sFA, (**d**) PVA-CHi, (**e**) PVA-CHi/FA, (**f**)PVA-CHi/sFA, (**g**) PVA-Alg, (**h**) PVA-Alg/FA, and (**i**) PVA-Alg/sFA nanofibers (Magnification: 5 kX, scale bar: 1 µm).

The neat blend fibers, FA-loaded composite fibers, and FA-sprayed composite fibers at the same quantities were successfully electrospun. The morphology of the fibers was characterized by SEM, as shown in Figure 2. To further analyze the diameter distributions of nanofibers, ImageJ software was used to determine the average diameter of 60 different and random nanofibers using SEM micrographs. Generally, the incorporation of FA decreases the average diameter of FA-loaded nanofibers. This could be due to the addition of FA into the polymer blend solutions, together with NaHCO_3_, as a result of which the viscosity decreased.

It was noticed that the PVA-Gel, PVA-Alg, and PVA-CHi nanofibers were bead-free and smooth. Unlike PVA-Alg, which included more fiber interactivity and coarser fibers, PVA/Gel and PVA-CHi did not contain interacting or coarser fibers. An interconnected fiber structure was revealed in both neat PVA-Alg and PVA-Alg/FA fibers. However, in the PVA-Alg/FA sample, the fiber thickness was even thinner, and the porosity increased slightly (208.2 nm, 48.97%) compared to the neat one due to the FA-NaHCO_3(aq)_ in neat PVA-Alg/FA fibers. As FA was sprayed on the fiber surfaces, morphology deteriorated (Figure 2i), and film-like structures formed on the fibers’ surfaces before the fibers dried. It is thought that this type of film formation is a consequence of the dissolution of the fiber with the solvent of the FA during the simultaneous process [10]. This heterogeneous appearance could also be an effect of controlled drug release. PVA-Gel fibers were dispersed and showed a tight structure with fewer fiber joints. On the other hand, FA-loading gave rise to larger fiber diameters and caused the fiber cross-section to adopt a flat form. After FA-loading, a partial film surface also formed, resulting from the viscosity decreasing due to the addition of the NaHCO_3_ (Figure 2b). As seen in Figure 2c, the simultaneous process facilitated the formation of FA beads on the PVA-Gel fibers’ surfaces, and/or layers, but the web morphology was destroyed as in the PVA-Alg/sFA and PVA-Gel/FA samples (Figure 2b,f). The SEM micrographs of the PVA-CHi nanofibers in a random spaghetti structure show that knotty fiber structures formed in some regions through a spontaneous process. Moreover, the fiber thickness increased from 96.26 nm to 101.86 nm, which occurred in parallel to porosity decline (Table 3).

**Figure 3:**
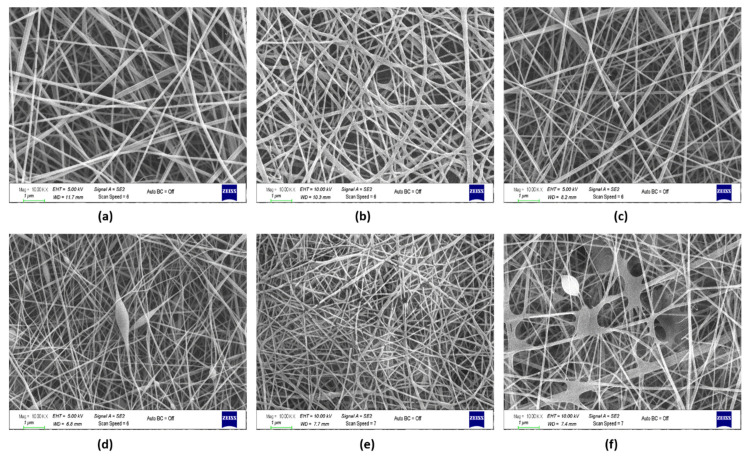
(**a**) SEM image of PVA-Gel–CHi, (**b**) PVA-Gel–CHi/FA, (**c**) PVA-Gel–CHi/sFA, (**d**) PVA-Alg–CHi, (**e**) PVA-Alg–CHi/FA, and (**f**) PVA-Alg–CHi/sFA nanofibers (Magnification: 10 kX, scale bar: 1 µm).

PVA-Gel–CHi fibers have a uniform shape and are randomly oriented. As FA was incorporated into the polymer solution, the interaction of the fibers increased and a rough structure was formed. The same situation was not observed when adding FA via the electrospraying method (Figure 3c). Decreasing the viscosity due to the addition of the NaHCO_3_ caused a different web structure. PVA-Gel–CHi/sFA fibers were almost the same as the neat fibers. These samples showed almost no fiber entanglement. Nevertheless, the average fiber diameter of the neat PVA-Gel–CHi sample decreased from 96.15 nm to 76.82 nm (Table 3). This could be because, during the e-spray process, the FA and polymer solutions dissolve in each other before reaching the collector.

The PVA-Alg–CHi web had a bead-on-string structure and thin and continuous fibers (Figure 3d). The PVA-Alg–CHi/FA web included discontinuous fibers and had a non-uniform web morphology, but not a bead-on-string structure. The addition of FA increased the fiber interaction and fiber breakdown due to increase secondary interactions in the electrospinning process (Figure 3e) (Li et al., 2012). In the PVA-Alg–CHi/sFA web, a PVA-Alg/sFA film formed as a web. The film formation occurred for the same reasons as in PVA-Alg/sFA.

### 3.2. Chemical Structure of the Hybrid Nanofibers

**Figure 4:**
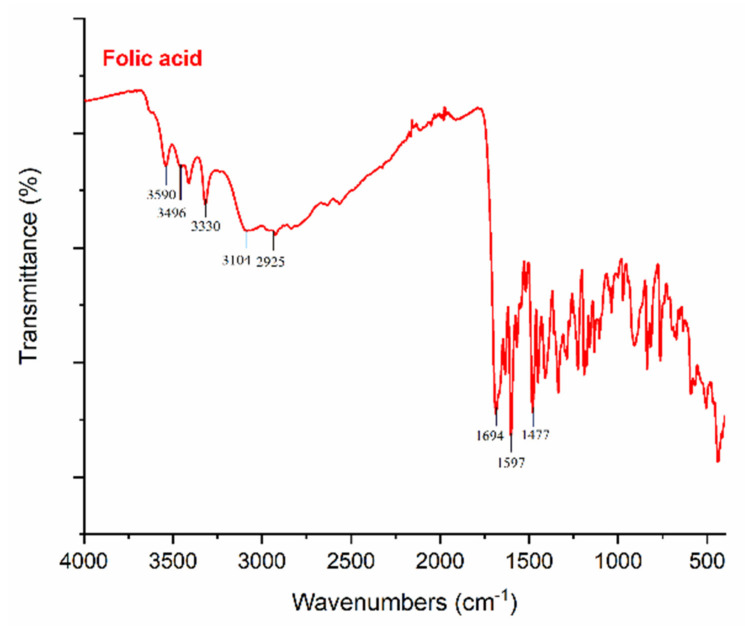
FT-IR spectra of folic acid.

**Figure 5:**
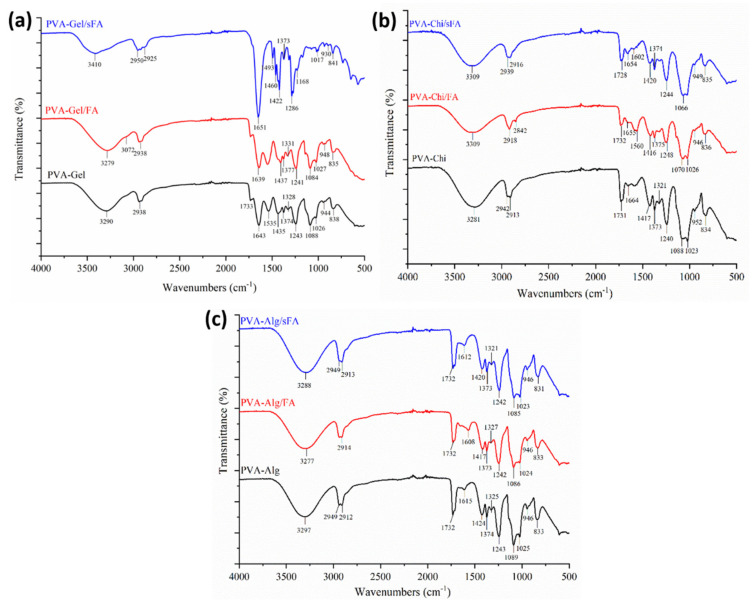
FT-IR spectra of binary nanofibers, (**a**) shows comparison of PVA-Gel/sFA, PVA-Gel/FA, and PVA-Gel, (**b**) shows spectra of PVA-Chi/sFA, PVA-Chi/FA, and PVA-Chi, and (**c**) shows spectra of PVA-Alg/sFA, PVA-Alg/FA, and PVA-Alg.

**Figure 6:**
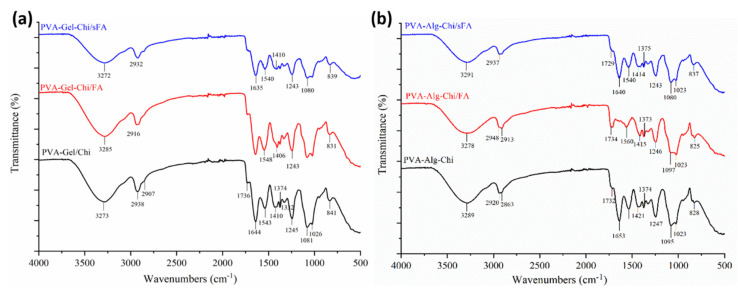
FT-IR spectra of ternary nanofibers (**a**) shows comparison of PVA-Alg/sFA, PVA-Alg/FA, and PVA-Alg, (**a**) shows spectra of PVA-Gel-Chi/sFA, PVA-Gel-Chi/FA, and PVA-Gel-Chi, and (**b**) shows spectra of PVA-Alg-Chi/sFA, PVA-Alg-Chi/FA, and PVA-Alg-Chi.

FT-IR spectra were acquired to investigate the physical interactions between polymer matrices and FA particles, as illustrated in Figure 4, Figure 5 and Figure 6. It was determined that FA particles were successfully sprayed onto the surface of PVA-Gel nanofibers due to the intense and clearly defined C=O stretching of folic acid at 1651 cm^−1^. The band of the (–OH) group occurs at 3290 cm^−1^ in PVA-Gel fibers. The bands at 2938 and 2913 cm^−1^ are from the –CH_2_ stretching vibration. The typical absorption peaks of gelatin are mostly due to peptide bonds (–CONH) with amide I–III vibrations. The peak at 1643 cm^−1^ (amide-I) is associated with –C=O stretching vibration, while the peak at 1535 cm^−1^ (amide-II) is related with N-H bending and C-H stretching vibration. The (amide-III) peak was recorded at 1243 cm^−1^. Further, 1435 cm^−1^ (–CH_2_ bending), 1374 cm^−1^ (C–H wagging), 1088 cm^−1^ (–C–O–C), and 837 cm^−1^ (C–C) stretching were obtained. The disappearance of specific peaks in the FT-IR spectra of PVA-Gel/FA (Figure 5a) indicates that FA was loaded into the nanofibers. It could imply that the FA is uniformly spread throughout the blended nanofiber.

The peaks at 3590 cm^−1^, 3496 cm^−1^, and 3330 cm^−1^ are linked with the (–OH) groups of folic acid in the crystalline structure. Moreover, the characteristic peaks of folic acid in the broad band between 3101 and 2400 cm^−1^ are caused by (–OH) groups of glutamic acid [35]. It has been found that the –NH stretching peak in the structure of the pterin ring disappeared in the broad band; however, the peak can be seen at 3101 cm^−1^ [36]. The (–C=O) group, related to pterin structure, can be observed at around 1694 cm^−1^ [35]. The peaks at 1635 cm^−1^ ((–C=N) stretching) and 1597 cm^−1^ belong to the bending of (–CONH_2_) [37], and 1477 cm^−1^ is related to the (–C=C) stretching of phenyl and pterin rings.

In PVA-CHi fibers, the band of the (–OH) group appears at about 3281 cm^−1^, related to –OH and –NH stretching vibrations. The FTIR spectra of the PVA/chitosan fibers (Figure 6b) show characteristic peaks of PVA and chitosan, with the exception of those associated with the ionization of the major amino groups of chitosan [38]. These peaks are signals at 1417 cm^−1^ and 1560 cm^−1^. The symmetric deformation of –NH_3_^+^ groups is responsible for the formation of the peak around 1560 cm^−1^, while the peak at 1417 cm^−1^ refers to carboxylic acid and those at 1321 cm^−1^ and 1240 cm^−1^ are connected to C-H vibration [39]. The peaks at 1731 cm^−1^ are typical of carboxylic acid dimers. The results are consistent with the literature [40].

The typical peaks of the PVA-Alg nanofiber were identified at 3297 cm^−1^, 2949 cm^−1^, 2912 cm^−1^, 1732 cm^−1^, and 1089 cm^−1^, attributed to –OH groups, asymmetric and symmetric –CH stretching, and –CO stretching, respectively (Figure 5c). The characteristic peaks of the electrospun PVA-Alg/FA nanofibers were detected at 3277 cm^−1^, 2949 cm^−1^, 2914 cm^−1^, 1732 cm^−1^ and 1086 cm^−1^, and are related to –OH groups, asymmetric and symmetric –CH stretching, and CO stretching, respectively. Moreover, the peaks at 1615 cm^−1^ and 1424 cm^−1^, resulting from asymmetric and symmetric (–COO) stretching, in the PVA-Alg fibers are slightly shifted to 1608 cm^−1^ and 1417 cm^−1^ in the PVA-Alg/FA fibers.

The characteristic peak of the carboxyl group of alginate, the amide group of chitosan, and the overlapping PVA peaks are similar to those in the PVA-Gel–CHi and PVA-Alg–CHi nanofibers (Figure 6a,b).

### 3.3. Thermal Properties and Weight-Loss of Nanofibers and Folic Acid

**Figure 7:**
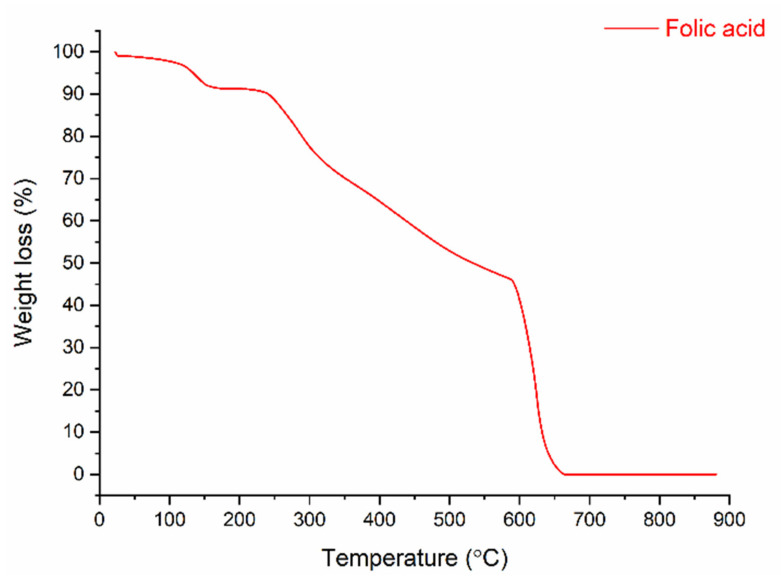
TGA thermogram of folic acid.

Figure 7 shows the thermogram of folic acid, which reveals that the bioactive powder was decomposed into four stages. In the first stage, the weight-loss of 7.54% was caused by removing moisture at temperatures from 100 to 170 °C. The 2.34% weight-loss in the second stage is related to the degradation of glutamic acid in the FA structure (between 170 and 240 °C) [41]. A slower degradation behavior occurred in the 240–600 °C range, with a weight-loss of about 49%. This was due to the degradation of the functional groups, which are pterin and p-amino benzoic acid (Janković, 2010). In the last stage, the 41.3% weight-loss was connected to the decomposition of pyrolysis products formed in the second and third steps.

**Figure 8:**
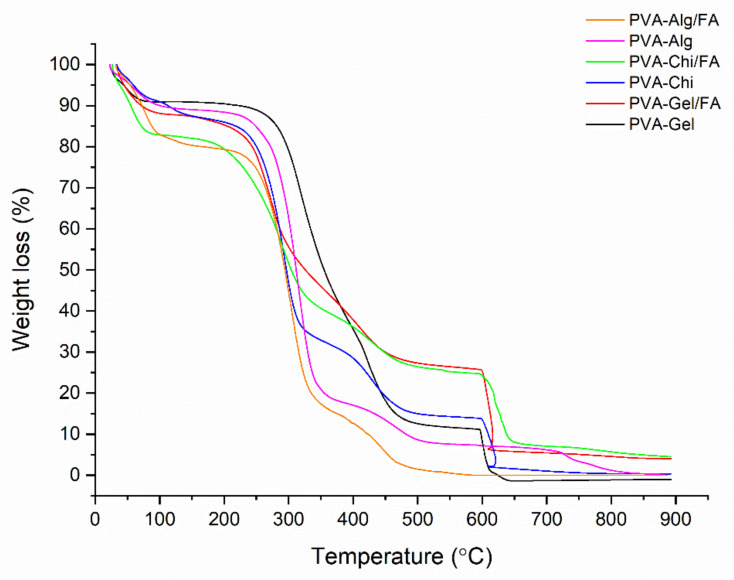
TGA thermogram of nanofibers.

TGA was used to assess the thermal stability of the nanofibers (Figure 8). Three main stages are seen in the TGA curves, which are linked to solvent and humidity being trapped inside (30–110 °C) the nanofiber samples; the second stage is connected to degradation of the polymers between 280 °C and 450 °C and the third stage is due to the degradation of the pyrolysis products of the polymers, which are formed during the polymer degradation stage under a N_2_ atmosphere [10,42]. The second weight-loss phase, related to the decomposition of the polymers, was composed of two steps according to the binary polymer mixture. The decomposition curves of PVA-Gel, PVA-Alg and PVA-CHi suggest two different decomposition behaviors. The first decomposition was related to the removal of the side group from the macromolecule chain. The last was related to the main macromolecule chain scission. Except for PVA-Alg/FA and PVA-Alg/FA, all samples gave pyrolysis products due to the inert atmosphere (N_2_ condition). After changing the atmosphere from N_2_ to O_2_ at 600 °C, all samples lost mass, except PVA-Alg/FA and PVA-Alg/FA, resulting from the alginate decomposition behavior in the inert atmosphere. Alginate does not create pyrolysis products when it decomposes under inert condition [43].

The decomposition of pure PVA has been discussed in the literature [31]. The thermal decomposition of the PVA mixture was hardly different from that of the pure PVA polymer, especially the last part of the curve. The first part of the polymer decomposition curve was also changed by adding FA molecules into the polymers. FA addition decreases the polymer decomposition speed due to its protective properties. This situation implies that FA could be used as an anti-oxidant in protective polymers during processing at high temperatures. FA’s conservation properties did not emerge in the case of the alginate polymers. The decomposition speed of the alginate mixture was not changed by adding FA due to the behavior of alginate decomposition in the inert atmosphere. The beginning of the first polymer decomposition stage was begun at lower temperatures by adding FA molecules. This resulted from the decomposition behavior of the FA materials [10,44]. The early decomposition of the FA molecules caused energy absorption, similar to the antioxidant running mechanism [45]. This energy absorption process preserves and delays the main polymers’ decomposition.

### 3.4. In Vitro Release Test and Kinetics

**Figure 9:**
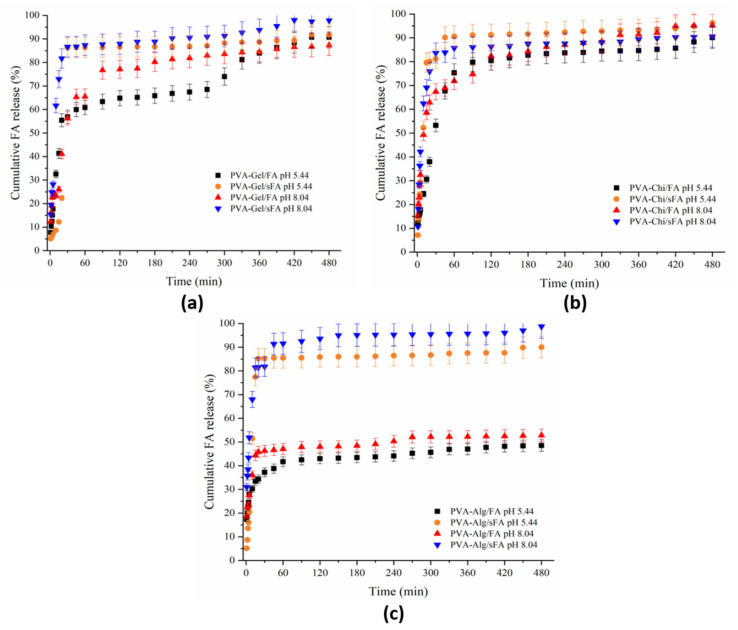
UV-Vis release of binary nanofibers (**a**) release behavior of PVA-Gel/FA at pH 5.44, and 8.04, and PVA-Gel/sFA at pH 5.44 and 8.04, (**b**) release behavior of PVA-Chi/FA at pH 5.44 and 8.04, and PVA-Chi/sFA at pH 5.44 and 8.04, while (**c**) shows release behavior of PVA-Alg/FA at pH 5.44 and 8.04, and PVA-Alg/sFA at pH 5.44 and 8.04 respectievly.

**Figure 10:**
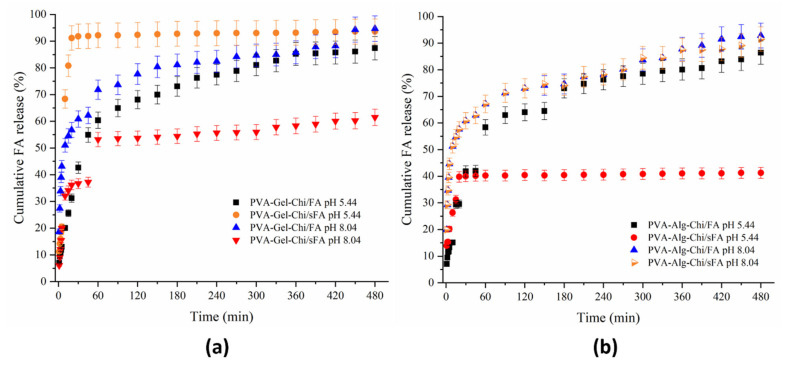
UV-Vis release of ternary nanofibers (**a**) shows release behavior of PVA-Gel-Chi/FA at pH 5.44 and 8.04, and PVA-Gel-Chi/sFA at pH 5.44 and 8.04, while (**b**) shows release behavior of PVA-Alg-Chi/FA at pH 5.44 and 8.04, and PVA-Alg-Chi/sFA at pH 5.44 and 8.04.

UV-Vis spectroscopy was utilized to analyze the FA release profile of the nanofibers, which assisted in revealing the structure–function connection of the electrospun fibers containing FA in the artificial acid (pH 5.44, 37 ℃) and alkaline (pH 8.04, 37 ℃) sweat solutions over 480 min. Figure 9 and Figure 10 depict the cumulative FA release rate profiles in the sweat solution samples, which were binary and ternary nanofibers containing FA. In Figure 9a, the release profiles of the PVA-Gel-sFA nanofibers in both acidic and alkaline media are similar, and they were released quickly in the first 30 min, then these samples continued to release FA slowly until 480 min. After 210 min, the FA release rate in alkaline media increased compared to acidic medium, and the maximum release value increased to almost 95%. The release behavior of the PVA-Gel/FA samples (in both acidic and alkaline medium) was partially different from that of PVA-Gel/sFA samples. These samples showed fast release behavior in alkaline medium for the first 30 min, while the fast release behavior in acidic medium continued until 90 min. Moreover, the amount of FA released in the alkaline medium was higher than in the acidic medium until 360 min. After this, the release rate increased in the acidic medium, and the cumulative FA release reached about 90%. It has been reported that the release media affects the polymer matrix, as well as the active drug [46].

It could be said that the fast release of FA from the samples produced via the electro- spraying process depended on the physical bond between the FA molecules and the fiber surface. It can be seen from Figure 2c that FA molecules were deposited on the fiber surface. When this type of web is immersed in acidic or alkaline media, the media breaks down the weak physical bond between fiber and FA molecules. The fast release of the samples in which FA was embedded was related to the depositing of FA on the fiber surface. Because of this, the release ratio was lower than that in the other samples produced via electrospraying. During the controlled release period, the release speed of the samples produced via electrospinning was higher than that of the others due to the removing of the embedded FA from inside the fiber.

The release behaviors of PVA-CHi/FA samples were the same in both acidic and alkaline medium (Figure 9b). After 180 min, the FA release rate increased in alkaline medium, and therefore the samples showed the highest quantity of released FA at the end of the release study. The amount released in acidic medium remained at almost 90%. Until 90 min, the release behavior of PVA-CHi/sFA was different from that of PVA-CHi/FA. A higher FA release rate occurred in the acidic medium (around 93%), while a lower FA release rate was observed in alkaline medium (around 85%).

Nair et al. (2019) performed a release study of curcumin-loaded chitosan nanoparticles by simulating skin at two distinct pH values (pH 5 and pH 7.4) [47]. In this study, the change in curcumin release observed under two different pH conditions was associated with the pH-dependent swelling behavior of chitosan. The swelling of chitosan allows the release medium to penetrate the polymer matrix and act as a plasticizer, which transforms the glassy polymer into a more rubbery structure, leading to increased drug release from the nanoparticle structure [48]. The release profiles of PVA-CHi/FA samples in both acidic and alkaline media are similar. Similarly, the release profiles of PVA-Kit/sFA in acidic and alkaline media are similar to each other. The PVA-CHi/FA samples released FA at 90% and 95% in acidic and alkaline media, respectively, due to the dissolution of PVA and chitosan. Mafenite acetate-loaded PVA-chitosan nanofibers were found to release 50% of the drug in the web structure within the first 10 min in the PBS medium [49].

The release rates of FA from the PVA-Alg/sFA nanofibers were similar in acidic and alkaline media (Figure 9c). PVA-Alg/sFA samples showed a fast release rate in the first 60 min, and then controlled release after this. It can be observed that the amount of FA released in these media during the analysis was over 90%. These findings are in agreement with the literature. Lutein-loaded sodium alginate/PVA nanofibers were found to achieve very fast lutein release, and cumulative release was completed in 7 min, at almost 92% [20]. The fast release in the first few minutes is explained by the hydrophilic character of alginate and the PVA composition. Arthanari et al. (2016) noted that gatifloxacin-loaded PVA-sodium alginate nanolyphs did not display a fast burst release tendency in the PBS (pH 7.4) for the first 1 h, but gatifloxacin was released (95% in total) during the test period [50]. The PVA-Alg/FA nanofibers in acidic and alkaline media were also similar, with almost 50% of FA released. PVA-Alg/FA had a fast release rate in the first 60 min. Unlike PVA-Alg/sFA samples, the PVA-Alg/FA samples’ FA release rate increased after 240 min. The PVA-Alg samples had the same release behavior as in PVA-Gel.

As seen Figure 10a, the ternary mixture of the PVA-Gel–CHi/FA sample had the same release profile in both acidic and alkaline media. These samples displayed fast release in the first 60 min. After this time, the release rate increased in a controlled manner. The release behaviors of PVA-Gel–CHi/sFA samples differed from those of PVA-Gel–CHi/FA samples. In addition, the release behaviors of PVA-Gel–CHi/sFA samples in acidic and alkaline media vary from each other. These samples showed fast release rates in alkaline medium in the first 60 min, and the release amount reached 60% in a controlled manner. In acidic medium, the samples showed fast release in the first 30 min and this was sustained in a controlled manner.

Apart from the PVA-Alg–CHi/sFA nanofiber structure in the acidic medium, the release rates of the other three webs were similar in both acidic and alkaline media (Figure 10b). For PVA-Alg–CHi/sFA nanofibers, the FA released in acidic medium was considerably low compared to the alkaline medium (41%). This could be related to the homogeneity of the electrospraying process. Furthermore, PVA-Alg–CHi/sFA exhibited a controlled release behavior after 30 min, while the other three samples showed gradual release to almost 90%. The PVA-Alg–CHi/FA samples showed little difference compared to the other two samples in the alkaline media.

Crosslinkers are often utilized to prepare PVA and/or gelatin-based scaffolds with physicochemical characteristics, and the obtained scaffolds are almost identical to healthy tissues. Besides this, drug release rates are advanced by crosslinkers. However, crosslinking reactions minimize the bio-based scaffolds’ in vivo degradation, and negatively affect host tissue responses [51,52]. In particular, gluteraldehyde (GA), which can be used as a crosslinker, can be considerably toxic to the body, and is typically restricted in many medicinal applications [53].

In the decision regarding the release mechanism, the choice of nanofiber matrix plays a critical role, dependent on degradation behavior and interaction with theurapeutics [54]. The diffusion mechanisms are based on both the polymer matrix properties and the processing variables, that is, whether they are Fickian or non-Fickian [55]. On the other hand, the solution matrix is more suitable for therapeutics, as these are dispersed in the electrospun fibers, resulting from the release rate and fiber morphology at which Fickian diffusion predominates. If a matrix shows considerably slow drug release, the Higuchi model (Power Law) is most often used to explain the diffusion-driven release. However, polymer degradation usually plays a substantial role in drug release, as is the case with PVA, in addition to release due to diffusion, when the matrix is biodegradable. The Korsmeyer–Peppas kinetic model typically defines such mixed release mechanisms [54].

The release profiles of the different nanofibers in two distinct release media correlated the best with different mathematical models (Table S1). The PVA-Alg–CHi/sFA nanofibers (in pH 5.44) gave the best results with zero-order, first-order, and Hixson–Crowell kinetic models. Generally, the Korsmeyer–Peppas kinetic model is used more than other models (Table S1). Moreover, the “n exponential factor” was found to be n < 1 and 0.45 < n < 0.89 for the nanofibers. For matrices in cylindrical form, the Fickian diffusion mechanism and signs of non-Fickian or abnormal transport are evident at 0.45 < n < 0.89. It was observed that both the change of release media and the use of different polymer matrices affects the release mechanism. It was found that the change in pH in the mechanism of FA release from the nanofibers obtained by the e-spraying method did not have a significant effect on the “n exponential factor”. However, the release of FA from nanofibers obtained by electrospinning was partially changed, depending on pH.

### 3.5. Entrapment Efficiency (EE, %) and Loading Capacity (LC, %)

The entrapment efficiency (EE, %) and also loading capacity (LC, %) have been calculated in the manuscript [56]. These values have been determined by the non-volatile FA structure using a UV-Vis spectrophotometer. The drug solubility of electrospun fibers influences the entrapment efficiency and the loading capacity of FA-loaded and FA-sprayed nanofibers (Table S2).

### 3.6. Cytotoxic Effects of Nanofibers by MTT Assay

**Figure 11:**
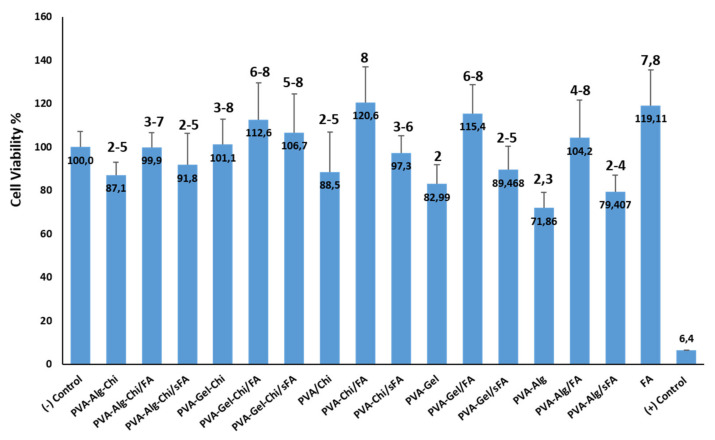
Effects of all nanofibers on cell viability of L929 cells, by MTT assay.

The hydrophilic character of these composite fibers, which may assist in connecting the desired cells to the surfaces of the scaffolding, thus enables cell development and the improvement of viability, which is desirable in the context of applications of tissue engineering [57]. The proliferation of cells seeded on electrospun FA-loaded and FA-sprayed composite and pure nanofibers was evaluated via MTT assay after 24 h of treatment in L929 cells. The MTT results indicate that not all nanofiber scaffolds showed considerable toxicity, since their viability was no less than 70% (Figure 11) [34]. All PVA-based composite fibers with FA could be widely utilized in wound dressing and tissue engineering fields due to their ability to support cell function. The results prove that the addition of FA into PVA-based nanofibers enhances cell growth and supports cell proliferation. FA-loaded bicomponent composite fibers promoted cell proliferation during the MTT assays. In this regard, the blending process is more efficient than simultaneous processes. This could be related to the excess alcohol from the FA solution affecting the nanofibers.

The PVA-Gel/FA, PVA-CHi/FA, and PVA-Alg/FA samples acheved cell viability values of 115.4 ± 13.1, 120.6 ± 16.4, and 104.2 ± 17, respectively. PVA-CHi/FA fibers had the highest cell viability value of 120.6 ± 16.4.

Folic acid plays a crucial role in cell growth and repair, assisting wound healing via the biosynthesis of nucleic acids [58,59]. It is pointed out that FA can be converted into active tetrahydrofolic acid owing to the response of folic acid reductase in vivo. As a major provider of a carbon unit, tetrahydrofolic acid plays a key role in purine and pyrimidine synthesis and in amino acid mutual transformation; the absence of this in vivo vitamin may affect the transmission of the carbide unit and may affect nucleic acid synthesis bad amino acid metabolism, which are necessary to proliferate, grow and develop cells. The presence of FA in cell division throughout the wound healing process improves the healing rate due to its effect on growing cell division [60].

All the electrospun composite fibers consisting of FA exhibited more cell viability (%) compared to pure ones. This can be explained by the fact that FA has a high affinity for the folate receptor on the L929 cells [38]. Furthermore, PVA-based nanofibers have been noted as excellent drug carriers in a matrix, due to their non-toxic and biocompatible features. Therefore, PVA is utilized for a large range of medicinal applications, especially in its fibrous form, through these characteristics [61,62,63].

The ECM structure of fibroblast, osteoblast, chondrocyte and endothelial cells are the same in micro and nanofibers arranged in a random fibrous mat, which allows for quick regeneration and differentiation [64]. In recent years, various wound dressings have been developed and evaluated with similar cytotoxicity results to ours. Kalalinia et al. (2021) developed vancomycin (VCM)-loaded hybrid chitosan/poly ethylene oxide (Chi/PEO) nanofibers by blend-electrospinning [65]. There are no harmful effects of any group, according to the findings of the Alamar Blue cytotoxicity tests performed on HDFs. Çetmi et al. (2019) fabricated microalgal extract-loaded PCL nanofibers, and MTT cell growth assays revealed that the new fibers enhance the growing of cardiovascular cells by contributing significantly to cell proliferation [66]. Similarly, carbon nanofiber/gold nanoparticle (CNF/GNP)-conductive nanofibers used as scaffold have been produced by Nekounam et al. (2020) using two strategies: e-spraying simultaneously with electrospinning and blending electrospinning methods [67]. In this study, the MTT assays performed on MG63 cells did not show any toxicity, and all obtained nanofibers showed biocompatibility with cells.

These PVA-based nanofibers not only exhibited good biocompatibility in vitro, but were also found to possess many preferable wound/tissue engineering applications. In sum, PVA-based FA scaffolds offered the best nanofibers for cell development and are appropriate candidates for tissue engineering and wound healing.

According to the SNK analysis, all nanofibers ranked between the positive control (1) and PVA-CHi/FA (8), the cell viability of which was higher than FA. FA promotes the growth of cells because its cell viability values are greater than negative. PVA-Gel and PVA-Alg nanofibers are in the same group (2). The PVA-Alg sample is also placed in the third group. PVA-Alg–CHi, PVA-CHi, PVA-Gel/sFA and PVA-Alg–CHi/sFA nanofibers are in the same groups (2, 3, 4, and 5). Moreover, PVA-Alg–CHi/FA are in the same groups (3, 4, 5, 6, and 7) as the negative control. The PVA-CHi/FA, PVA-Gel/FA, PVA-Gel–CHi/FA and PVA-Gel–CHi/Sfa samples are considered to be more effective in terms of cell viability than others, based on the SNK analysis. The MTT assay results of all nanofibers show they would be acceptable for use as biocompatible materials in accordance with ISO 10993-5.

## 4. Conclusions

In the current study, FA-loaded and FA-sprayed PVA-based nanofibers were successfully fabricated via two distinct approaches: electrospinning and simultaneous electrospinning and electrospraying processes. The SEM micrographs indicate that spraying FA onto web layers and/or surfaces affects the nanofiber’s surface morphology. On the other hand, FA-loading did not change the morphology without phase separation, owing to the compatibility of FA with polymer matrices. The PVA gel kit samples achieved the highest porosity of 54.3 ± 3.8%, while PVA gel kit/sFA samples had the lowest fiber diameter (76.8 ± 2 nm) based on ImageJ software measurement. Based on FT-IR analysis, the resultant FA-sprayed composite fibers included the characteristic peaks of both polymer and FA. Furthermore, the TGA thermogram confirmed FA can be used as an antioxidant agent to delay the decomposition of polymer blends in the processing stage. PVA-CHi/FA webs had the highest release value of 95.2% in alkaline media, whereas PVA-Gel–CHi/sFA webs released the most FA (92%) in acidic media. The highest entrapment efficiency value was determined as 94.82 ± 4.7% for the PVA-Gel/sFA sample, while the lowest entrapment efficiency was determined for PVA-Alg/FA at 50.66 ± 2.5%. The loading capacity values of FA-loaded and FA-sprayed nanofibers were lower than 3%. Meanwhile, the MTT assays of PVA-CHi/FA samples showed that they had the highest cell viability, at 120.6 ± 16.4. Moreover, the FA release mechanism from the nanofibers was either Fickian or non-Fickian (abnormal transport) due to the change in matrices. The cytotoxicity studies of L929 cells performed via MTT assay displayed good biocompatibility, and even these hybrid nanofibers supported cell proliferation. In view of the results, nanofibrous PVA-based materials containing FA samples are recommended for tissue scaffold applications.

## Figures and Tables

**Table 1:** Process parameters for electrospinning.

Fiber Type	Polymer Ratio (*v*/*v*)	Voltage (kV)	Distance (mm)
PVA-Gel	4/1	27	75
PVA-CHi	4/1	24	97
PVA-Alg	4/1	26	90
PVA-Gel–CHi	3/1/1	26	78
PVA-Alg–CHi	3/1/1	26	78

**Table 2:** Kinetic models used to calculate FA release from the nanofibers.

Kinetic Model	Equation
Zero-order	$Q_{t}=k_{0}t+Q_{0}$
	Q_t_: Drug concentration at time t
	Q_0_: Initial drug concentration
	k_0_: Zero order rate constant
First-order	$\log Q_{t}=\log Q_{0}-\mathrm{kt}\;/2303$
	Q: Drug concentration released at time t
	Q_0_: Initial drug concentration
	k: First order rate constant
Higuchi (Power Law)	$Q_{t}=k_{h\;}t^{1/2}$
	Q: Drug concentration at time t
	k_h_: Higuchi release rate constant
Hixson-Crowell	${\;Q}_{0}{}^{1/3}-{\;\mathrm{Qt}}^{\frac{1}{3}}=k_{h}$
	Q_0_: Initial drug amount
	Qt: Drug amount at time t
	k: Hixson–Crowell rate constant
Korsmeyer–Peppas	$\frac{\mathrm{Qt}}{Q\infty }={\mathrm{kt}}^{n}$
	Qt: Drug concentration at time t
	Q∞: Equilibrium drug concentration in the release medium
	Qt/Q∞: Drug fraction in the release medium at time t
	k: Release rate constant
	n: Diffusional exponent

**Table 3:** Average diameter and porosity of the fibers.

Sample	Average Diameter of Fibers (nm)	Porosity (%)
PVA-Gel	101.07 ± 30.0	46.55 ± 2.30
PVA-Gel/FA	115.23 ± 39.8	34.88 ± 4.93
PVA-Gel/sFA	145.33 ± 31.65	43.46 ± 1.16
PVA-CHi	96.26 ± 25.3	48.93 ± 0.82
PVA-CHi/FA	92.66 ± 23.9	52.09 ± 3.47
PVA-CHi/sFA	101.86 ± 36.02	42.97 ± 1.22
PVA-Alg	269.51 ± 70.25	44.31 ± 2.35
PVA-Alg/FA	208.2 ± 47.32	48.97 ± 2.66
PVA-Alg/sFA	158.17 ± 48.55	43.97 ± 7.49
PVA-Gel–CHi	96.15 ± 29	54.3 ± 3.79
PVA-Gel–CHi/FA	106.94 ± 32.13	47.15 ± 1.03
PVA-Gel–CHi/sFA	76.82 ± 20	52.2 ± 2.14
PVA-Alg–CHi	85.96 ± 30.4	52.2 ± 2.13
PVA-Alg–CHi/FA	84.45 ± 29.07	51.21 ± 0.94
PVA-Alg–CHi/sFA	123.53 ± 66.16	51.34 ± 2.01

## Data Availability

The data presented in this study are available on request from the corresponding author.

## Supplementary Materials

The following are available online at https://www.mdpi.com/article/10.3390/polym13203594/s1. Table S1: Regression values of the FA-loaded and FA-sprayed nanofibers, Table S2: The entrapment efficiency (%) and loading capacities (%) of all nanofibers.
