# Supplementary material for: Fabrication and Characterization of Electrospun Folic Acid/Hybrid Fibers: In Vitro Controlled Release Study and Cytocompatibility Assays
Source: Polymers (Basel). 2021 Oct 19;13(20):3594. doi: 10.3390/polym13203594 (PMC8537833; doi:10.3390/polym13203594)
Supplement: Supplementary file 1 [file polymers-13-03594-s001.zip › polymers-1408434-supplementary.pdf]

Research Article (Supplementary data)

Fabrication and Characterization of Electrospun Folic Acid/Hybrid Fibers: In Vitro Controlled Release Study and Cytocompatibility Assays

Fatma Nur Parın[1*], Sana Ullah[2], Kenan Yıldırım[1], Motahira Hashmi[2], Ick Soo Kim[2*]

[1]Faculty of Engineering and Nature Science, Department of Polymer Materials Engineering, Bursa Technical University, Mimar Sinan Campus, Turkey
[2] Graduate School of Medicine Science and Technology, Department of Science and Technology, Division of Smart Material, Shinshu University Ueda Campus, Japan

* **Correspondence:**
Dr. Fatma Nur Parin
nur.parin@btu.edu.tr

Prof. Dr. Ick Soo Kim
Kim@shinshu-u.ac.jp

Table S1. Regression values of the FA-loaded and FA-sprayed nanofibers.

	Kinetic models					
**Sample ID**	**Zero-order**	**First-order**	**Hixson-Crowell**	**Higuchi**	**Korsmeyer-Peppas**	**"n" value**
PVA-Gel/FA (pH 5.44)	0.71	0.88	0.84	0.84	0.97	0.61
PVA-Gel/FA (pH 8.04)	0.81	0.88	0.86	0.93	0.87	0.32
PVA-Gel/sFA (pH 5.44)	0.94	0.93	0.93	0.82	0.95	0.22
PVA-Gel/sFA (pH 8.04)	0.44	0.71	0.61	0.63	0.96	0.37
PVA-Chi/FA (pH 5.44)	0.77	0.86	0.83	0.9	0.94	0.43
PVA-Chi/FA (pH 8.04)	0.7	0.94	0.88	0.84	0.98	0.51
PVA-Chi/sFA FA (pH 5.44)	0.46	0.64	0.99	0.66	0.99	0.86

PVA-Chi/sFA FA (pH 8.04)	0.43	0.55	0.5	0.63	0.99	0.87
PVA-Alg/FA (pH 5.44)	0.65	0.7	0.69	0.83	0.95	0.15
PVA-Alg/FA (pH 8.04)	0.57	0.6	0.59	0.76	0.9	0.18
PVA-Alg/sFA (pH 5.44)	0.87	0.91	0.9	0.94	0.78	0.46
PVA-Alg/sFA (pH 8.04)	0.74	0.92	0.79	0.87	0.96	0.31
PVA-Gel-Chi/FA (pH 5.44)	0.77	0.92	0.87	0.92	0.98	0.55
PVA-Gel-Chi/FA (pH 8.04)	0.82	0.95	0.93	0.94	0.89	0.31
PVA-Gel-Chi/sFA (pH 5.44)	0.95	0.99	0.98	0.96	0.99	0.35
PVA-Gel-Chi/sFA (pH 8.04)	0.67	0.73	0.71	0.86	0.94	0.35
PVA-Alg-Chi/FA (pH 5.44)	0.77	0.91	0.86	0.92	0.96	0.52
PVA-Alg-Chi/FA (pH 8.04)	0.76	0.95	0.9	0.89	0.94	0.34
PVA-Alg-Chi/sFA (pH 5.44)	0.98	0.98	0.98	0.96	0.93	0.35
PVA-Alg-Chi/sFA (pH 8.04)	0.74	0.93	0.88	0.88	0.94	0.34

Table S2. The entrapment efficiency (%) and loading capacities (%) of all nanofibers.

Sample ID	Entrapment efficiency (%)	Loading capacity (%)
PVA-Gel/FA	89.03 ±4.4	1.53 ±0.08
PVA-Gel/sFA	94.82 ± 4.7	1.53 ±0.08
PVA-Chi/FA	93.11 ± 4.6	2.34 ±0.12
PVA-Chi/sFA	93.35 ± 4.6	2.34 ±0.12

PVA-Alg/FA	50.66 ± 2.5	1.12 ±0.05
PVA-Alg/sFA	94.36 ±4.72	1.12 ±0.05
PVA-Gel-Chi/FA	91.06 ±4.55	2.53 ±0.13
PVA-Gel-Chi/sFA	77.56 ±3.87	2.53 ±0.13
PVA-Alg-Chi/FA	89.62 ±4.48	2.82 ±0.14
PVA-Alg-Chi/sFA	66.38±3.32	2.82 ±0.14
